# Effects of Counterion on the Formation and Hydration Behavior of α-Form Hydrated Crystals (α-Gels)

**DOI:** 10.3390/gels9120928

**Published:** 2023-11-25

**Authors:** Kenichi Sakai, Shuri Nishimoto, Yuki Hirai, Kyosuke Arakawa, Masaaki Akamatsu, Keisuke Tanaka, Toshiyuki Suzuki, Hideki Sakai

**Affiliations:** 1 Department of Pure and Applied Chemistry, Faculty of Science and Technology, Tokyo University of Science, 2641 Yamazaki, Noda 278-8510, Chiba, Japank-arakawa@rs.tus.ac.jp (K.A.); hisakai@rs.tus.ac.jp (H.S.); 2 Research Institute for Science and Technology, Tokyo University of Science, 2641 Yamazaki, Noda 278-8510, Chiba, Japan; makamatsu@tottori-u.ac.jp (M.A.); suzutosh@nikkolgroup.com (T.S.); 3 Department of Chemistry and Biotechnology, Faculty of Engineering, Tottori University, 4-101 Koyama-Minami, Tottori 680-8552, Tottori, Japan; 4 R&D Center, Nikko Chemicals. Co., Ltd., NIKKOL GROUP, 3-24-3 Hasune, Itabashi 174-0046, Tokyo, Japan; tanakeis@nikkolgroup.com

**Keywords:** α-form hydrated crystal, α-gel, lamellar gel, humidity, hydration, water sorption, quartz crystal microbalance with dissipation monitoring (QCM-D), surfactant, counterion

## Abstract

α-Form hydrated crystals form a lamellar gel in which the alkyl chains of the amphiphilic molecules are hexagonally arranged within bilayers below the gel–liquid crystal phase transition temperature. In practice, the lamellar gel network with excess water is called an “α-gel”, particularly in the cosmetics industry. In this study, the hydration or water sorption of amphiphilic materials in water vapor was assessed using a humidity-controlled quartz crystal microbalance with dissipation monitoring (QCM-D) technique. The amphiphilic materials used in this study were hexadecyl phosphate salts neutralized with L-arginine (C16P-Arg), CsOH (C16P-Cs), KOH (C16P-K), and NaOH (C16P-Na). Small- and wide-angle X-ray scattering measurements revealed that C16P-Arg and C16P-Cs yielded α-form hydrated crystals. Humidity-controlled QCM-D measurements demonstrated that C16P-Arg and C16P-Cs more readily underwent hydration or water sorption than C16P-K and C16P-Na. The key conclusion is that the significant hydration ability of C16P-Arg and C16P-Cs promotes the formation of the corresponding α-form hydrated crystals.

## 1. Introduction

Mixtures of surfactants, long-chain alcohols (higher alcohols), and water can yield lamellar gels. Similar to a lamellar liquid crystal, a lamellar gel comprises bilayers that sandwich water layers at equal intervals. The structural difference between a lamellar gel and lamellar liquid crystal lies in the fact that the mobility of the alkyl chains forming the bilayers is strictly limited in the lamellar gel, but is high in the lamellar liquid crystal because the alkyl chains are in a molten state. In other words, a lamellar gel is formed below the gel-liquid crystal phase transition temperature (*T*_C_), whereas a lamellar liquid crystal is formed above *T*_C_ [1,2].

In some cases, the alkyl chains of the surfactants and long-chain alcohols are arranged hexagonally within the bilayers below *T*_C_. This characteristic bilayer structure is called an “α-form hydrated crystal” and, in practice, the lamellar gel network with excess water is called an “α-gel”, particularly in the cosmetics industry [3,4]. The excess water is not incorporated between the bilayers, but is instead localized between colloidal domains consisting of the α-form hydrated crystals. α-Gels are generally in a non-equilibrium or metastable state, similar to emulsions. α-Gels form the base for many personal care products such as hair conditioners and hand creams, owing to their high viscosity and excellent water retention ability [5]. α-Gels can be regarded as a kind of physical gel, and the formation and collapse of the network structure of these gels can be manipulated by external factors. The formation of the network structure is driven by domain-to-domain interactions with colloidal dimensions. Characterizing the domain-to-domain interaction is important for industrial applications because this interaction is a key factor controlling the rheology and stability of α-gels [5,6,7,8,9,10].

**Figure 1:**
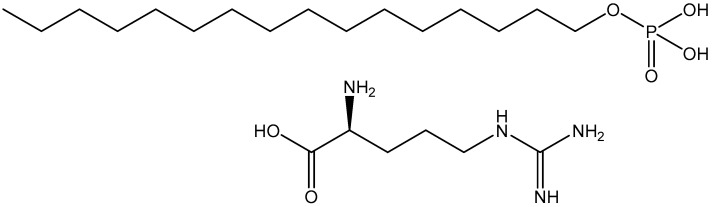
Chemical structures of C16P and Arg.

As mentioned earlier, the co-addition of long-chain alcohols with surfactants is a general method of preparing α-form hydrated crystals (α-gels). However, the addition of long-chain alcohols is not always necessary. For example, we previously demonstrated that neutralization of the salt of hexadecyl phosphate by L-arginine (C16P-Arg, Figure 1) yielded the α-form hydrated crystal structure over a wide range of temperatures and concentrations without adding long-chain alcohols [11,12]. Furthermore, the α-form hydrated crystal was formed by simply mixing C16P-Arg and water at room temperature without heating [11]. Wang and Marangoni [13] reviewed potential applications of α-form hydrated crystals (α-gels) in the food industry. Glycerol derivatives such as monoglycerides, polyglycerol fatty acid esters, sodium stearoyl lactylate, and propylene glycerol monostearate are frequently used in such applications. These surfactants also yield the α-form hydrated crystal structure without adding long-chain alcohols, although this structure is generally in a metastable state and gradually transforms into a thermodynamically more stable coagel structure. Emulsions and foams are stabilized as a result of the formation of the α-form hydrated crystal structure at oil/water and air/water interfaces [14,15,16].

In water, the alkyl chains of surfactants interact with each other via the van der Waals interaction below the Krafft temperature. This results in the crystallization of alkyl chains with orthorhombic or monoclinic packing arrangement, leading to the formation of hydrated crystals or coagels. However, the presence of bulky hydrophilic parts (i.e., headgroups and counterions of surfactants) may disrupt the packing arrangement and transform the closely packed arrangement into the metastable hexagonal structure (i.e., α-form hydrated crystal) even in the absence of long-chain alcohols. It is postulated, therefore, that the hydration of the components plays a key role in the formation of the α-form hydrated crystal.

**Figure 2:**
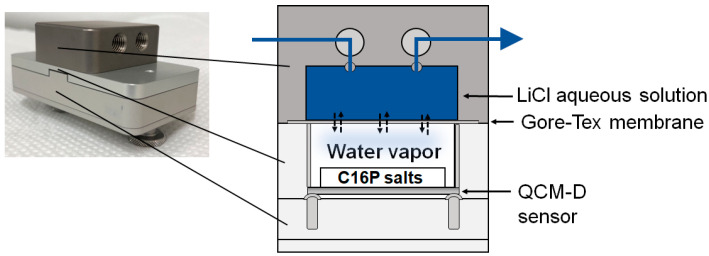
Schematic of QCM-D humidity module.

The hydration of materials or sorption of water from the gas phase can be assessed using a quartz crystal microbalance with dissipation monitoring (QCM-D) technique equipped with a humidity module. A schematic of the humidity module is shown in Figure 2. The QCM-D sensor is separated from an electrolyte solution by a Gore-Tex^®^ membrane, and the humidity of an air-filled compartment over the sensor is controlled at a fixed temperature. The humidity-controlled QCM-D technique enables simultaneous measurements of the frequency and energy dissipation shifts of materials on a QCM-D sensor surface induced by humidity changes in the gas phase. The frequency shift primarily indicates a change in mass, whereas the dissipation shift indicates a change in the viscoelastic properties of the materials. Hence, this technique can be used to monitor humidity-induced changes in the mass and viscoelastic properties of materials coated on sensor surfaces. The humidity-controlled QCM-D technique has been used to monitor the hydration or water sorption of various materials such as biopolymers [17,18,19,20], mesoporous particles [21], and surfactants [22,23,24].

In this study, we demonstrate that hydration is a key factor in determining whether C16P salts can yield α-form hydrated crystals in water. A humidity-controlled QCM-D technique is used for this purpose owing to its high sensitivity to water sorption. This technique has been used for monitoring the phase transition of surfactants in water. For example, the phase transition between a solid and a lyotropic liquid crystal was monitored in a few surfactant systems [22,24]. To the best of our knowledge, however, the hydration-induced formation of α-form hydrated crystals has not been studied. The physicochemical properties of α-gels are generally influenced by the size of their colloidal domains. Our present study allows us to evaluate the interactions between amphiphilic materials and water, being important for understanding the formation mechanism of α-form hydrated crystals or α-gels. The C16P salts used are C16P-Arg, C16P-Cs (cesium salt), C16P-K (potassium salt), and C16P-Na (sodium salt). It is expected that some of these materials will form α-gels and others will form hydrated crystals or coagels. Therefore, comparing the hydration behavior of these materials will contribute to elucidate the formation mechanism of α-gels. The formation of α-form hydrated crystals is confirmed via small- and wide-angle X-ray scattering (SWAXS) analysis.

## 2. Results and Discussion

### 2.1. Characterization of α-Form Hydrated Crystal (α-Gel)

**Figure 3:**
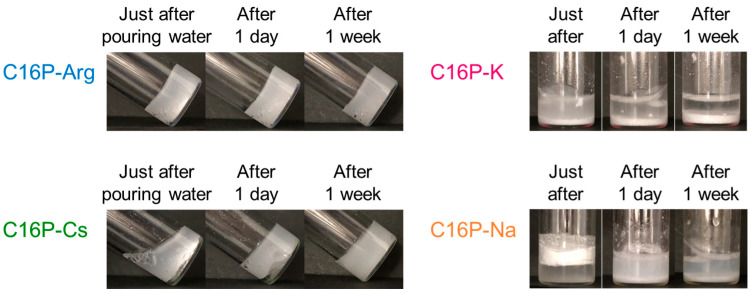
Visual appearance of the neutralized C16P samples at 0.2 mol/kg in water. These pictures were taken at 25 °C immediately after pouring water onto the neutralized C16P samples, and 1 day and 1 week thereafter.

Figure 3 shows the visual appearance of the neutralized C16P samples treated with water. The concentration of neutralized C16P was fixed at 0.2 mol/kg. This concentration corresponds to 10 mass% of the C16P-Arg system. These photographs were taken at 25 °C immediately after pouring water onto the neutralized C16P samples and 1 day and 1 week thereafter. As demonstrated in our previous studies [11,12], C16P-Arg yielded a highly viscous white sample. Visually, no phase separation was observed in this sample. For C16P-Cs, phase separation was observed immediately after pouring water on the material. However, phase separation was not observed one week thereafter; instead, the sample became uniformly white and highly viscous. In contrast, a white gel with a uniform phase was not formed by the C16P-K and C16P-Na systems over the entire investigation period. Plausibly, the solid phase observed was a coagel or hydrated crystals of the C16P salts formed below their Krafft temperature [2].

**Figure 4:**
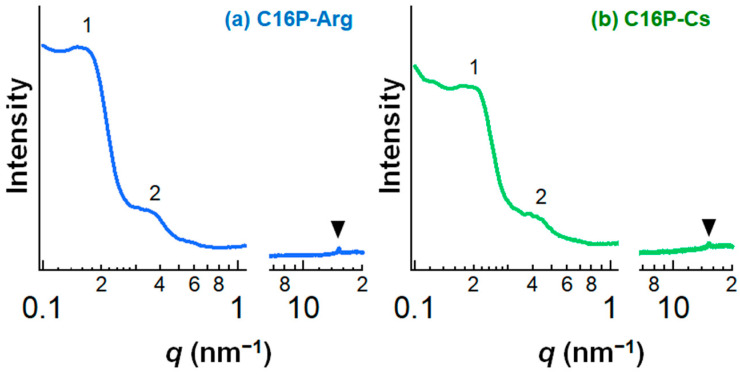
SWAXS data for the (**a**) C16P-Arg and (**b**) C16P-Cs systems. The concentration of the neutralized C16P salts was 0.2 mol/kg in water. These systems were equilibrated at 25 °C for 2 weeks after preparation. The measurements were performed at a constant temperature of 25 °C. In this figure, the first and second peaks observed in the small-angle region are represented as “1” and “2”, and the peak observed in the wide-angle region is represented as a triangle symbol.

SWAXS was used to confirm the formation of the α-form hydrated crystals [2]. Figure 4 shows the SWAXS profiles of the C16P-Arg and C16P-Cs systems. Repeated peaks were observed for the two systems in the small-angle region at a scattering vector (*q*) ratio of 1:2, indicating the formation of a lamellar bilayer. A sharp peak was also detected in the wide-angle region at *q* = ca. 15 nm^−1^, indicating that the alkyl chains within the bilayers adopted an hexagonal arrangement. The SWAXS data confirm that C16P-Arg and C16P-Cs can yield α-form hydrated crystals, as expected.

The first peak observed in the small-angle region was used to calculate the lamellar *d*-spacing (*d*) using the following equation.

*d* = 2π/*q* (1)

The smaller *q* value obtained for the C16P-Arg system (*q* = 0.18 nm^−1^) compared to that for the C16P-Cs system (*q* = 0.20 nm^−1^) indicates the larger *d*-spacing of the C16P-Arg system; *d* = 35 nm for C16P-Arg and 31 nm for C16P-Cs. The larger *d*-spacing of the C16P-Arg system compared to that of the C16P-Cs system was also suggested when we characterized the corresponding α-form hydrated crystals prepared at high temperatures above the *T*_c_ (see Supplementary Information, Figure S1). Iwata and Aramaki [25] demonstrated the effect of counterion species on the lamellar *d*-spacing of α-form hydrated crystals. The counterion strongly bound to the headgroup reduces the electrostatic repulsion between the lamellar bilayers, and hence yields a smaller *d*-spacing. In our systems, electrical conductivity measurements suggest that the degree of counterion binding was lower for the Arg salt than for the Cs salt in aqueous solution (Supplementary Information, Figure S2). Therefore, the larger *d*-spacing of the C16P-Arg system may arise from the relatively weak binding of Arg^+^ to the phosphate headgroup that forms the bilayers.

Differential scanning calorimetry (DSC) was used to confirm the *T*_c_ of each α-form hydrated crystal. The results are shown in Figure S3 (Supplementary Information). An endothermic peak was detected at 52 °C for the C16P-Arg system and at 48 °C for the C16P-Cs system, respectively. These temperatures correspond to the *T*_c_ of each α-form hydrated crystal formed by C16P-Arg and C16P-Cs in water. We confirmed, therefore, that the α-form hydrated crystals were formed just by pouring water on the C16P salts at 25 °C (below *T*_c_) without heating. At present, we have no clear interpretation of the difference in *T*_c_ observed for the two systems. It will be necessary to assess interactions between counterion-counterion and/or counterion-headgroup, affecting the packing arrangement of alkyl chains forming the bilayer in the α-form hydrated crystal structure.

### 2.2. Hydration or Water Sorption from Gas Phase

**Figure 5:**
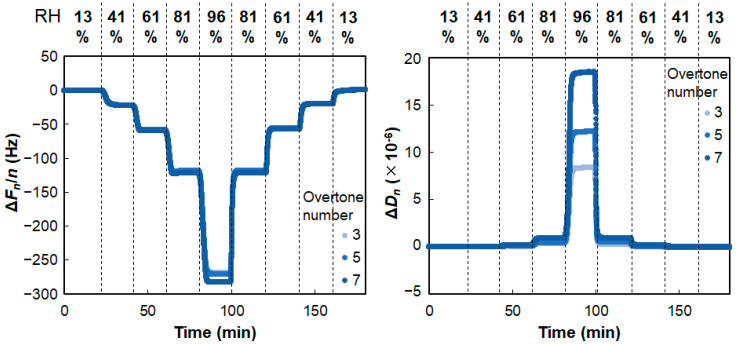
QCM-D responses of C16P-Arg system. The relative humidity was controlled by injecting aqueous LiCl solutions of different concentrations: 13% ([LiCl] = 18.5 mol/kg), 41% ([LiCl] = 10.0 mol/kg), 61% ([LiCl] = 7.0 mol/kg), 81% ([LiCl] = 4.0 mol/kg), and 96% ([LiCl] = 1.0 mol/kg). The relative humidity (RH) values were calculated based on the water activity (*a*_w_) [26]: RH = *a*_w_ × 100. The measurements were performed at a constant temperature of 25 °C.

Because the formation of α-form hydrated crystals (α-gels) was expected to be influenced by the hydration of the constituents, the capability of the neutralized C16P salts to undergo hydration or sorb water from the gas phase was assessed. Figure 5 shows the changes in the frequency (Δ*F_n_*/*n*) and energy dissipation (Δ*D_n_*) at different overtones (*n*). These measurements were performed by injecting aqueous LiCl solutions of different concentrations into a QCM-D humidity module to control the relative humidity in an air-filled compartment over a sensor coated with the neutralized C16P salts (see Figure 2). The results for the C16P-Arg system are shown in Figure 5, as a typical example.

In the increased humidity (wetting) process, the frequency decreased, whereas the dissipation increased. The observed change in frequency was more obvious than that in dissipation. These changes occurred rapidly in response to changes in the humidity. In the decreased humidity (i.e., drying) process, on the other hand, the frequency increased and the dissipation decreased. Again, these changes occurred rapidly. Importantly, the steady values of the frequency and dissipation were almost identical in the wetting and drying processes at each overtone. This suggests that wetting and drying of C16P-Arg are reversible.

**Figure 6:**
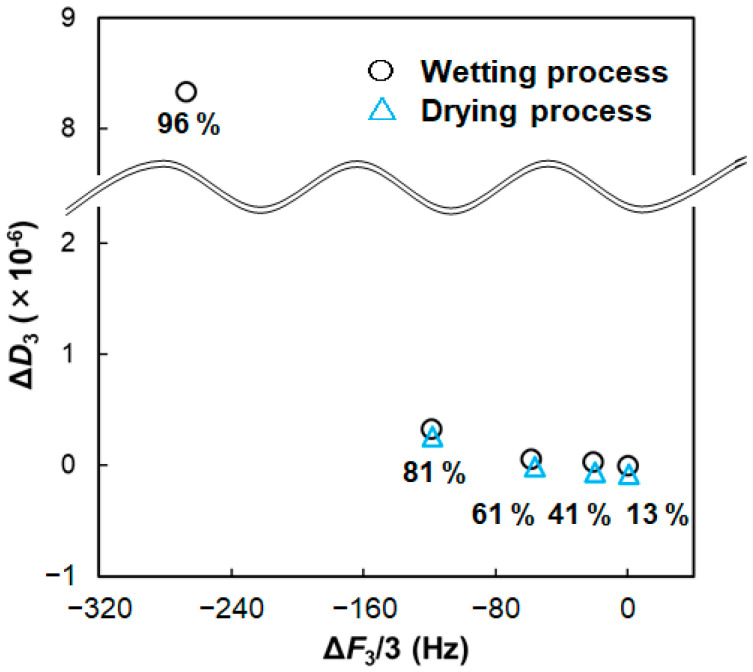
Δ*F_n_*/*n*–Δ*D_n_* plots based on the QCM-D responses in Figure 5 (C16P-Arg).

The steady frequency and dissipation values for the C16P-Arg system are plotted in Figure 6. The frequency changes primarily reflect a change in the mass of the sensor surface, whereas the dissipation changes reflect a change in the viscoelastic properties of the material. The energy dissipation (*D*) is defined as:

*D* = *E*_lost_/2π*E*_stored_ (2)

where *E*_lost_ is the lost or dissipated energy, and *E*_stored_ is the energy stored in the oscillator [27]. Hence the Δ*F_n_*/*n*–Δ*D_n_* plot reveals the relationship between the mass and viscoelastic properties of the materials on the sensor surface. As shown in Figure 6, increased humidity resulted in decreased frequency and increased dissipation; however, the dissipation changes were very small (<1 × 10^−6^) in the relative humidity range of 13–81%. These observations indicate the following two phenomena: (i) C16P-Arg was hydrated in the humidity range, inducing an increase in the mass, and (ii) hydrated C16P-Arg was still elasticity-dominant (or rigid).

The QCM-D responses were measured for the other three systems (C16P-Cs, C16P-K, and C16P-Na), as shown in Figure S4 (Supplementary Information). The results obtained for the C16P-K and C16P-Na systems were qualitatively similar to those obtained for the C16P-Arg system. In the C16P-Na system, however, the scattering of the frequency and dissipation data tended to increase, and instability of the dissipation values was observed during the drying process. In the C16P-Cs system, interestingly, extremely slow responses were observed at a relative humidity of 61% during wetting. This may reflect the slow swelling observed in Figure 3. Additionally, an unexpected increase in the frequency was observed at a relative humidity of 96%. Although we have no clear interpretation of this result, water may undergo condensation in the partially hydrated C16P-Cs sample because of its extremely slow swelling nature. Reversible QCM-D responses during the wetting and drying processes were observed for the C16P-Cs system below a relative humidity of 61%.

**Figure 7:**
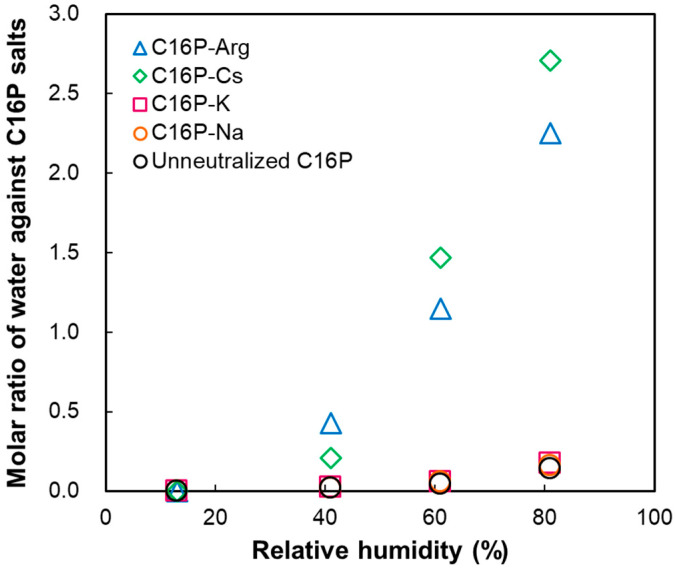
Amount of water coupled with the neutralized C16P salts. The data obtained for the unneutralized C16P are also shown for comparison.

The Sauerbrey equation [28] is used to calculate the change in the mass of a material on a sensor surface (Δ*m*):

Δ*m* = −*C*·Δ*F_n_*/*n* (3)

where *C* is a constant (=0.177 mg/(m^2^·Hz)). This calculation can be applied to the low dissipation region. Figure 7 shows the molar ratio of water molecules bound to the neutralized C16P salts during the wetting process as a function of the relative humidity (13–81%). The C16P salts were drop-cast; therefore, it was possible to estimate the amount of C16P salt on the surface of the QCM-D sensor. This allowed us to calculate the molar ratio of the water molecules bound to the C16P salts mentioned above. Increased humidity resulted in an increased molar ratio for all systems investigated. This increased molar ratio was particularly evident for the C16P-Arg and C16P-Cs systems. As mentioned earlier, these two systems can form α-form hydrated crystals in water. It is suggested, therefore, that the significant hydration ability of these C16P salts contributes to the formation of α-form hydrated crystals.

We previously investigated the thermal behavior of water in the C16P-Arg system [12]. That study showed that the water bound to the hydrophilic parts of C16P-Arg (i.e., headgroup and counterion) did not freeze even below 0 °C. In the C16P-Arg system, measurements of the melting enthalpy of water further suggested the saturation of bound water (i.e., non-freezing water) at a water concentration of approximately 20 mass%. This concentration corresponds to the molar ratio of water/C16P-Arg = 6.9, which was higher than that of the C16P-Arg system estimated at a relative humidity of 81%. Plausibly, hydration or water sorption from the gas phase occurs at the hydrophilic parts of C16P-Arg, and the water molecules in the α-form hydrated crystal act as non-freezing water.

Björklund and Kocherbitov [22] reported hydration-induced phase transitions of surfactants. Humidity-controlled QCM-D measurements revealed that the phase transition from a solid to a lyotropic liquid crystal resulted in drastic changes in the frequency and dissipation at an appropriate water activity. Additionally, phase transitions between lyotropic liquid crystals were detected as inflection points in the QCM-D responses. In the present case, drastic changes in the frequency and dissipation were observed (Figure 6) at a high relative humidity of 96%. These changes indicate that significant hydration or water sorption occurs at such a high relative humidity, and the hydrated C16P-Arg becomes more viscous. Although the significant increase in the dissipation makes it difficult to apply the Sauerbrey equation, the observed frequency shift roughly corresponds to the molar ratio of water/C16P-Arg = 5. This value is close to the saturation level of bound water (i.e., non-freezing water), as mentioned earlier. We suggest, therefore, that the hydration or water sorption that occurred at such high relative humidity may yield “interlayer water” or “excess water” [12], inducing increased energy dissipation of the α-form hydrated crystal.

At present, the exact reason why the hydration abilities of C16P-Arg and C16P-Cs are greater than those of C16P-K and C16P-Na is unknown. In our previous study [11], we hypothesized that the bulky counterions moderately suppressed the crystallization of the hexadecyl chains in water, leading to the formation of α-form hydrated crystals. Under this hypothesis, the larger counterions (Arg^+^ and Cs^+^) result in a greater hydration ability of the hydrophilic region within the lamellar bilayer, leading to the formation of α-form hydrated crystals. More recently, a detailed analysis using small-angle X-ray scattering indicated that the hexadecyl chains of C16P-Arg are interdigitated within the lamellar membrane [29]. The interdigitated structure may increase the distance between the counterions and facilitate hydration of the hydrophilic region. Further studies are necessary to understand the packing arrangement of the hexadecyl chains and the electron density of the headgroups with counterions.

## 3. Conclusions

The formation of α-form hydrated crystals (α-gels) was discussed from the perspective of hydration or water sorption from the gas phase. SWAXS measurements confirmed that the salts of hexadecyl phosphate neutralized by L-arginine (C16P-Arg) and CsOH (C16P-Cs) yielded the α-form hydrated crystal structure. The humidity-controlled QCM-D technique demonstrated hydration or water sorption by the C16P salts from the water vapor phase. The QCM-D measurements further demonstrated that C16P-Arg and C16P-Cs were more readily hydrated than C16P-K (potassium salt) and C16P-Na (sodium salt). We suggest, therefore, that the significant hydration ability of C16P-Arg and C16P-Cs is relevant to the formation of the α-form hydrated crystals. At a low relative humidity (less than 81%), hydration or water sorption from the gas phase occurs in the hydrophilic parts of the C16P salts, and the water molecules primarily act as non-freezing water. Plausibly, higher humidity (i.e., 96%) also yields “interlayer water” or “excess water,” inducing the increase in energy dissipation of the α-form hydrated crystals. The sorption and desorption behavior of water in α-gel systems affects the usability of cream products in the cosmetics industry. The findings of this study will be useful in such industrial applications.

α-Gels have been considered to be a non-equilibrium or metastable state. However, in some cases including our C16P-Arg and C16P-Cs systems, α-gels are spontaneously formed by simply mixing the corresponding materials with water. In such systems, water not only acts simply as a solvent, but also contributes to the formation of the α-form hydrated crystal structure as a component. Practically, α-gels have been studied by engineers or formulators in the fields of cosmetics and food industries. We expect that the industry and academia will continue to share knowledge about α-gels, and that the structure and function of α-gels will be further clarified.

## 4. Materials and Methods

### 4.1. Materials

C16P was provided by Nikko Chemical Corporation and used without further purification. Arg was purchased from Tokyo Chemical Industry Corporation. Aqueous solutions of NaOH (1 mol/dm^3^) and CsOH (50 mass%) were purchased from Fujifilm Wako Pure Chemical Corporation. KOH and LiCl were purchased from either Fujifilm Wako Pure Chemical Corporation or Tokyo Chemical Industry Corporation. All materials were used as received. The water used in this study was purified using either a Thermo Fisher Scientific Barnstead NANO Pure DIamond UV water purification system (Waltham, MA, USA) or a Merck Millipore Milli-Q^®^ Reference water purification system (Darmstadt, Germany).

### 4.2. Sample Preparation

C16P was neutralized with bases (Arg, CsOH, KOH, and NaOH) at a fixed molar ratio of 1:1.

In the low-temperature preparation method (corresponding to the results shown in Figure 3, Figure 4 and Figure S3), water was gently poured onto the neutralized C16P samples in a glass vial, and the system was equilibrated at 25 °C without stirring. The concentration of the neutralized C16P was adjusted to 0.2 mol/kg for a given total mass (the neutralized C16P + water) of 1.0 g.

In the high-temperature preparation method (corresponding to the results shown in Figure S1), the concentration of the neutralized C16P was also adjusted to 0.2 mol/kg for a given total mass (the neutralized C16P + water) of 1.0 g. The neutralized C16P samples were put in a glass vial and mixed using a vortex mixer for 90 s. Then, the samples were heated at 80 °C for 30 min. The vortex mixing and subsequent heating were repeated twice. Further mixing was performed thrice in the following combination; (i) heating at 80 °C for 1 h and (ii) centrifugal stirring for 1 h at a constant rotation speed of 2000 rpm using a Kokusan H11-NB centrifuge (Saitama, Japan). Finally, each sample was equilibrated for 5 weeks in an incubator set at a constant temperature of 25 °C.

For the QCM-D measurements, the neutralized C16P samples were freeze-dried under reduced pressure.

### 4.3. Measurements

SWAXS measurements were performed using an Anton Paar SAXSess camera (Graz, Austria) equipped with a PANalytical PW3830 laboratory X-ray generator, multilayer film Goebel mirror, block collimator, semi-transmissible beam stop, TCS120 temperature controller, and an imaging plate detector. The apparatus was operated at 40 kV and 50 mA using Cu-Kα X-rays (wavelength of 0.154 nm). The duration of X-ray irradiation was fixed at 20 min. Because of the translucent beam stop, the raw scattering data consistently include a reduced primary intensity at the scattering vector *q* = 0. All data were normalized to the same incident primary beam intensity for transmission calibration. The measurements were performed at a constant temperature of 25 °C.

QCM-D measurements were performed using either a Biolin Scientific Q-Sense Explorer or Analyzer (Gothenburg, Sweden). Silica-coated QCM-D sensors (QSX 303) were used for these measurements. Prior to each measurement, the sensor was cleaned using a Sun Energy SKB401Y-02 UV-ozone cleaner (Osaka, Japan) for 10 min, ultrasonicated for 30 min in an aqueous solution of sodium dodecyl sulfate (2 mass%), rinsed thoroughly with water, dried in a nitrogen flow, and finally cleaned using the UV-ozone cleaner again. The cleaned silica-coated QCM-D sensor was drop-coated with neutralized C16P samples dissolved in chloroform/methanol mixtures. Typically, the concentration of the neutralized C16P was adjusted to 2.4 mmol/dm^3^, and the solution (16 µL) was dropped on each QCM-D sensor. After drying under reduced pressure for 12 h, the sensor was set in a QCM-D humidity module (QHM 401) equipped with a Gore-Tex^®^ membrane. Aqueous LiCl solutions prepared at different concentrations were injected stepwise into the module to control the relative humidity in the air-filled compartment over the sensor. The flow rate of the LiCl solution was constant at 0.05 cm^3^/min. The measurements were performed at a constant temperature of 25 °C.

Electrical conductivity measurements of aqueous solutions of C16P-Arg and C16P-Cs were performed using a DKK-TOA CM25-R electrical conductivity meter equipped with a CT-57101C immersion-type conductivity cell (Tokyo, Japan). The measurements were performed at 60 °C.

DSC measurements were performed using a Rigaku DSC8230 calorimeter (Tokyo, Japan). The samples were introduced in an aluminum vessel and the vessel was then sealed. The measurement conditions were as follows; scanning rate = 1 K/min, scanning range = 25−80 °C, and sensitivity = 4.18 × 10^−4^ J/s (=0.1 mcal/s).

## Data Availability

The data presented in this study are available on request from the corresponding author. The data are not publicly available due to ongoing research using a part of the data.

## Supplementary Materials

The following supporting information can be downloaded at: https://www.mdpi.com/article/10.3390/gels9120928/s1, Figure S1: SWAXS data for the (a) C16P-Arg and (b) C16P-Cs α-form hydrated crystals prepared at high temperatures above the *T*_c_. Figure S2: Electrical conductivity of aqueous solutions of C16P-Arg and C16P-Cs measured at 60 °C. Figure S3: DSC data for the (a) C16P-Arg and (b) C16P-Cs systems. Figure S4: QCM-D responses of C16P-Cs, C16P-K, and C16P-Na systems.
