# Supplementary material for: Effects of Counterion on the Formation and Hydration Behavior of α-Form Hydrated Crystals (α-Gels)
Source: Gels. 2023 Nov 25;9(12):928. doi: 10.3390/gels9120928 (PMC10742572; doi:10.3390/gels9120928)
Supplement: Supplementary file 1 [file gels-09-00928-s001.zip › gels-2714338-supplementary.pdf]

Supplementary Materials

Effects of counterion on the formation and hydration behavior of α-form hydrated crystals (α-gels)

Kenichi Sakai[1, 2*], Shuri Nishimoto[1], Yuki Hirai[1], Kyosuke Arakawa[1], Masaaki Akamatsu[2, 3], Keisuke Tanaka[4], Toshiyuki Suzuki[2, 4], and Hideki Sakai[1, 2]

1. Department of Pure and Applied Chemistry, Faculty of Science and Technology, Tokyo University of Science
2. Research Institute for Science and Technology, Tokyo University of Science
3. Department of Chemistry and Biotechnology, Faculty of Engineering, Tottori University
4. R&D Center, Nikko Chemicals. Co., Ltd., NIKKOL GROUP

*Correspondence: k-sakai@rs.tus.ac.jp; +81 4 7124 1501 (2641 Yamazaki, Noda, Chiba 278-8510, JAPAN)

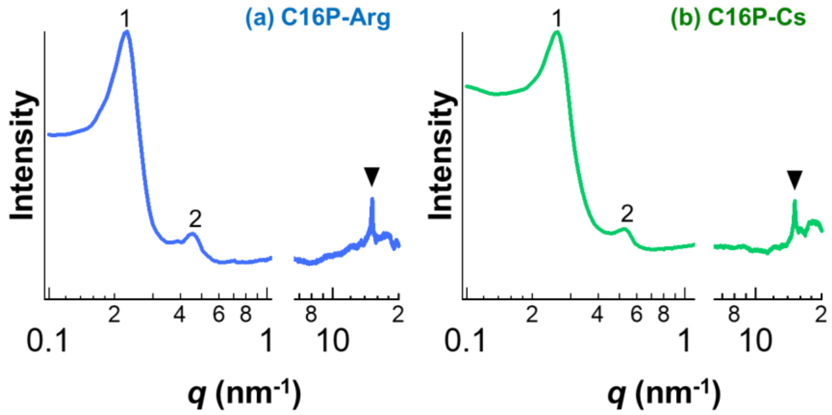

Figure S1: SWAXS data for the (a) C16P-Arg and (b) C16P-Cs α-form hydrated crystals prepared at high temperatures above the T_c. The SWAXS data calculate d = 27 nm (q = 0.23 nm^{-1}) for C16P-Arg and d = 24 nm (q = 0.26 nm^{-1}) for C16P-Cs, respectively.

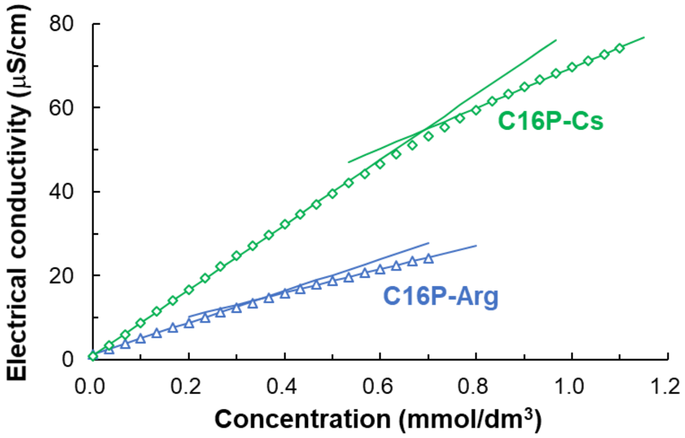

Figure S2: Electrical conductivity of aqueous solutions of C16P-Arg and C16P-Cs measured at 60 °C. The degree of counterion binding was estimated as 0.26 for C16P-Arg and 0.38 for C16P-Cs, respectively.

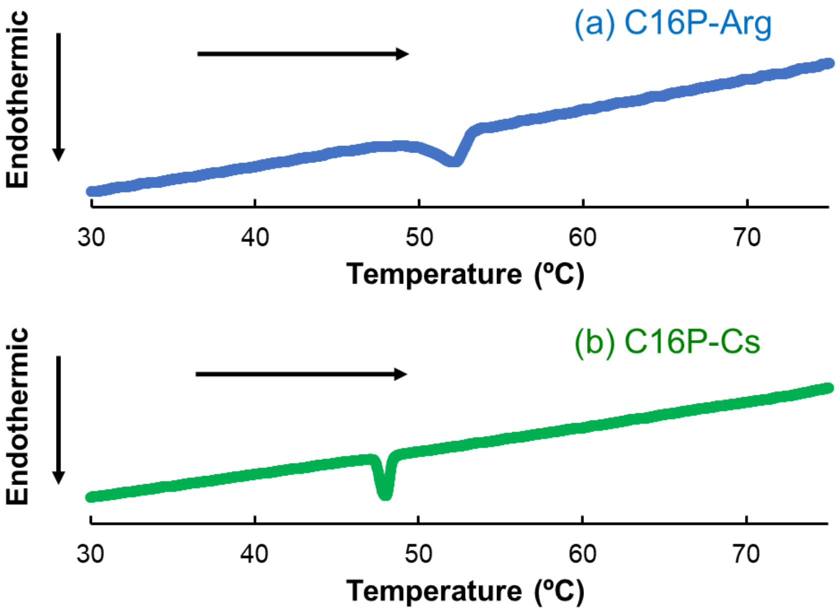

Figure S3. DSC data for the (a) C16P-Arg and (b) C16P-Cs systems. The concentration of the neutralized C16P salts was 0.2 mol/kg in water. These systems were equilibrated at 25 °C for 2 weeks after preparation.

(a) C16P-Cs

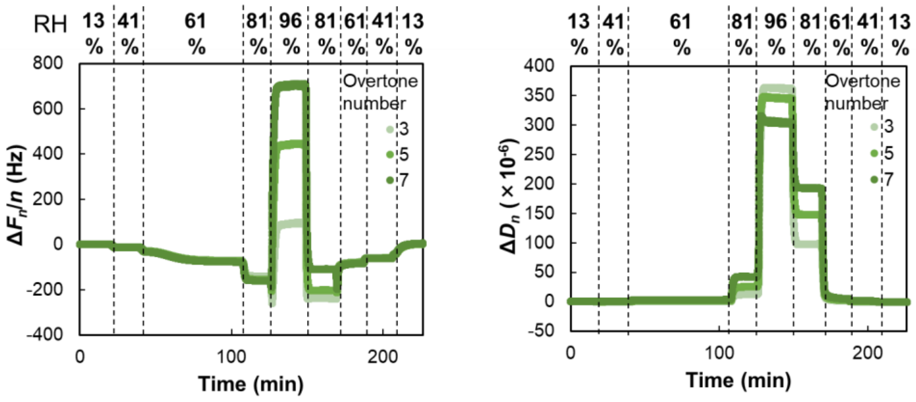

(b) C16P-K

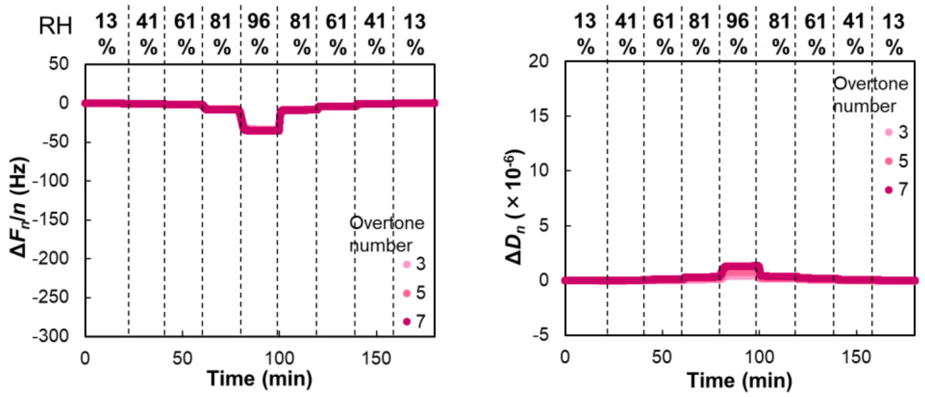

(c) C16P-Na

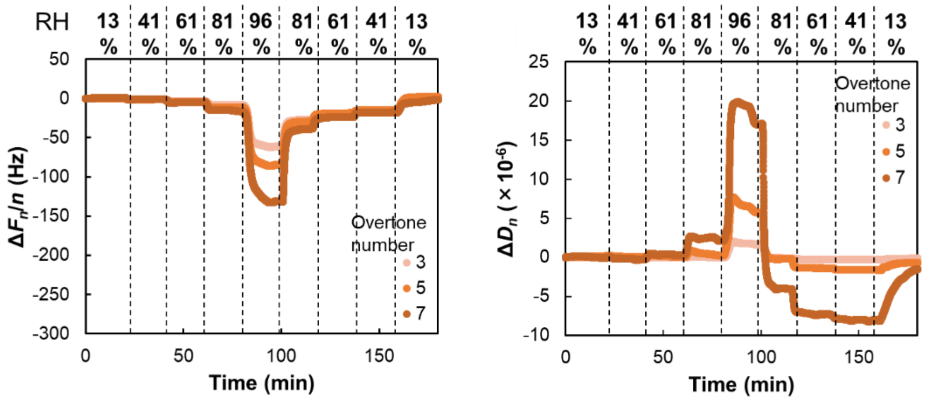

Figure S4: QCM-D responses of (a) C16P-Cs, (b) C16P-K, and (c) C16P-Na systems. The relative humidity was controlled by injecting aqueous LiCl solutions of different concentrations: 13% ([LiCl] = 18.5 mol/kg), 41% ([LiCl] = 10.0 mol/kg), 61% ([LiCl] = 7.0 mol/kg), 81% ([LiCl] = 4.0 mol/kg), and 96% ([LiCl] = 1.0 mol/kg). The relative humidity (RH) values were calculated based on the water activity (a_w): RH = $a_w \times 100$.
